# Unsupervised Process Anomaly Detection and Identification Using the Leave-One-Variable-Out Approach †

**DOI:** 10.3390/s25072098

**Published:** 2025-03-27

**Authors:** Jacob A. Farber, Ahmad Y. Al Rashdan

**Affiliations:** Department of Automation, Instrumentation, and Controls, Idaho National Laboratory, Idaho Falls, ID 83415, USA; ahmad.alrashdan@inl.gov

**Keywords:** anomaly detection, anomaly identification, leave-one-variable-out model, online monitoring, root cause analysis

## Abstract

Automated anomaly detection and identification can signal equipment issues and pinpoint causes in large-scale industrial systems. For systems with limited failure history, unsupervised machine learning methods can be utilized as they do not require past failures. This study introduces the leave-one-variable-out (LOVO) model, which masks one variable at a time to predict the others, learning underlying process correlations. Detection performance was assessed with synthetic and experimental data, while identification performance used only synthetic data due to its ability to generate labeled anomaly types. For detection using synthetic data, the LOVO model generally outperformed comparative models; while using experimental data, the comparative methods outperformed the LOVO model. However, the comparative methods required selecting a latent size, and these conclusions pertain to using the optimal size. In practice, it would not be feasible to always select the optimal value, and incorrect selections impacted performance. In contrast, the LOVO model does not require a latent space. For identification using synthetic data, the LOVO model was slightly outperformed in interpretability and repeatability but still demonstrated impressive results. These outcomes suggest that the LOVO model is an effective model and may be more easily implemented without the challenging tuning process of selecting a latent size.

## 1. Introduction

The operational reliability and safety of large-scale industrial systems rely heavily on the proper functioning of their individual components and process equipment. For systems featuring a significant number of such equipment, monitoring all this equipment can result in significant operation and maintenance costs. Examples of such systems include nuclear power plants, oil refineries, chemical plants, and water treatment facilities. One challenge is that the size of the systems may make it uneconomical to create system-specific automated monitoring approaches for every system. Thus, the approach to handling many faults and failures is either to have humans perform time-intensive inspections and examinations, or to be reactive in nature (i.e., wait until there are obvious indications of failure). While less time-intensive upfront, the reactive approach may provide only minimal lead time to procure parts and schedule repairs, and may result in minor faults cascading into larger system failures.

As a result of the scale of these systems, there is significant room for improved monitoring via the use of automated anomaly detection approaches. A common approach to anomaly detection for process equipment is to develop some type of mathematical model that predicts the behavior of some measured parameter, and then compare the predictions to the measured values. This can be achieved using either data-driven or physics-based methods. For the present effort, machine learning (ML) (i.e., data-driven) methods were selected over physics-based ones because ML methods are system-agnostic and thus rely purely on historical data to develop models. This means the same approach can be taken whether the equipment in question is pumps, valves, or heat exchangers. By contrast, physics-based methods require a substantial modeling effort in order to capture every part of the plant, and since many of those modeled systems will never experience an anomaly, even after decades of operation, no return on investment is obtained from modeling them.

Once an anomalous event has been detected, and if its cause is not immediately obvious, the next step is to identify the cause of the anomaly so that the condition can be corrected. This is currently a labor-intensive and costly process, made even more difficult by the size and complexity of large-scale systems. As an alternative, automated identification methods could help subject matter experts (SMEs) diagnose anomalies. This would reduce labor costs, enable more efficient responses to detected events, and prevent unexpected downtimes. The approach developed and tested in this study covers both detecting and identifying anomalies.

Most ML-based anomaly detection and identification approaches fall into one of two categories: supervised or unsupervised. The key difference is that in supervised learning, there must be labeled historical anomalies (for each type of anomaly if performing identification), which can be used to compare against new process data. Because this study focuses on large systems featuring a significant number of components, many of which seldom fail, the use of supervised approaches is not recommended, as there would be few labeled data on which to train the algorithms. Instead, this study focuses on unsupervised ML methods for anomaly detection and identification. These methods entail addressing the complex issue of automatically identifying an anomaly never before encountered by the algorithm. Given the broad spectrum of anomaly types, it is unlikely that a fully unsupervised approach could definitively isolate the issue to a single cause. Consequently, the goal is to provide information that can assist an SME in identifying the potential causes of the anomaly.

The objective of the present study is to detail the newly developed leave-one-variable-out (LOVO) anomaly detection and identification approach [1]. The LOVO model masks one variable at a time to predict the values of the other variables, and then assigns an anomaly status to new process data based on the prediction errors. The anomalous data then undergo the identification process (derived from the class of contribution-based identification approaches [2,3,4,5]), which aims to identify each variable’s contribution to the detected event. The contribution-based approaches and other related works are described in Section 2, and the LOVO approach is described in Section 3.

To quantify the results, it was necessary to have labeled process data containing both normal and anomalous behavior. The present study utilized a combination of synthetic and experimental data to assess detection performance. However, only synthetic data were used to assess identification performance due to the challenges of obtaining labeled experimental data with multiple examples across a range of different anomaly types. The synthetic data were modeled after spring–mass–damper (SMD) systems commonly found in mechanical engineering reference texts. The experimental data used were the open-source Skoltech Anomaly Benchmark (SKAB) dataset [6], which consists of a water circulation loop with a water tank, motor-driven pump, valves, and sensors. These datasets are detailed in Section 4. Using these datasets, assessment metrics for both detection and identification were first proposed (Section 5) and then implemented to generate results (Section 6).

In addition to presenting results for the LOVO approach, comparative results were also generated for other algorithms. Theses included principal component analysis (PCA), autoencoder (AE), and isolation forest (iForest) models, with the iForest model utilizing PCA for feature extraction. Notably, all of these methods employ the concept of a latent space, projecting the raw data onto a lower-dimensional space. A challenge with these methods is their sensitivity to the size of the latent space, as demonstrated in Section 6.

What is unique about the LOVO approach is that it does not require selection of the latent size, yet can offer comparable performance to other approaches, even when those approaches use their optimal latent size. This feature is particularly important for implementing comprehensive condition-monitoring programs in large-scale systems, for which it would be prohibitively expensive to manually review and tune every model used.

## 2. Related Work

Numerous methods of anomaly detection exist, and many review papers have covered them in detail (e.g., [7,8,9]). However, the present effort focuses on performing both unsupervised anomaly detection and identification, so this section centers around methods capable of both. A review of the literature revealed two main categories of methods that met the criteria: contribution-based and causality-based. Outside of data-driven methods, which are the focus of this study, physics-based and physics-informed methods also meet many of the criteria because they are capable of performing anomaly detection and identification without relying on data-driven techniques.

Contribution-based identification methods assume that the anomaly will cause significant deviations in its associated measurement values. Such methods aim to identify each variable’s contributions to the detected event. The contribution-based methods utilized in previous efforts employed either principal component analysis (PCA) or dynamic PCA (DPCA) models, which are commonly used to compress unordered and ordered (e.g., time-series) data, respectively. In contribution-based methods, PCA and DPCA models compress the data into a reduced-dimension representation, then reconstruct them to generate predictions of the data. This reduced-dimension representation is often referred to as the latent space, and its size is known as the latent size. For a given dataset, a model (called the undamaged equipment model) is trained to reconstruct data under anomaly-free operating conditions. The model can then be used to calculate an anomaly score, which serves as a measure of the degree of error between the input data and the reconstructed data. When this score exceeds a certain threshold, an anomalous event is considered to have been detected, and the identification process begins. At this stage, two types of contribution-based methods can be applied: contribution plot methods and reconstruction-based contribution methods.

In contribution plot methods, contributions are calculated directly from the anomaly score (for an example, see [10]). In general, the anomaly score is some function of the individual prediction error (often a sum of the squared errors) pertaining to each variable in the model. Thus, the contribution plot method simply decomposes the scalar anomaly score into error terms for each variable, and these terms can then be used for identification. However, this method can lead to misdiagnoses due to the “smearing effect” [3], which is the notion that, even for an anomaly that only affects a single variable (e.g., a sensor anomaly), the model may smear an error into multiple variables, due to the breakdown in the learned correlations.

**Figure 1:**
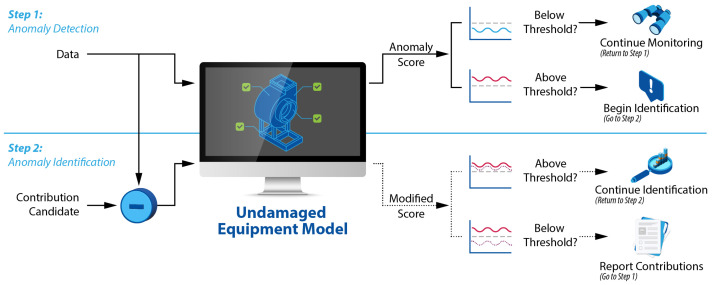
Schematic of reconstruction-based contribution methods.

To overcome this limitation, the reconstruction-based contribution method was developed [2,3,4,5]. Instead of simply decomposing the error, this method tries to recreate anomaly-free data that would lead to a reduced anomaly score. To accomplish this, the data are modified by subtracting a contribution from some or all the variables, then running the modified data through the same undamaged equipment model until the modified data’s anomaly score falls below the threshold. This process, shown in Figure 1, is called a reconstruction-based approach because it uses a reconstruction model (i.e., it takes measurement data as input, performs a dimensionality reduction, then tries to recover the input data from the reduced data) in an attempt to reconstruct normal operating data (i.e., data with a reduced anomaly score), rather than simply decomposing the anomaly score. This process is the same as that used with the developed LOVO approach.

Moving to the next category, causality-based identification methods assume that problems in upstream processes cascade down to their dependent processes, resulting in temporal misalignment between sensors in the upstream and the downstream processes. Previous research led to several approaches for assessing such temporal misalignment, including dynamic time warping [11,12], Granger causality [13], and transfer entropy [14]. This class of methods was not the focus of the present study, as such methods are often aimed at specific types of anomalies. For example, none of the aforementioned papers examined sensor anomalies, for which the idea of causality may be less appropriate.

Finally, physics-based and physics-informed methods leverage prior knowledge and first-principles models to detect and diagnose equipment problems. Although not the primary focus of this effort, it is worth discussing how these methods operate. These approaches include directly using physics-based models to detect behavioral changes and utilizing these models to identify deviations in behavior [15,16,17]. This framework is also commonly used in digital twins [18,19,20], which are virtual representations of physical objects or processes that are used for monitoring, control, and many other applications. Alternatively, simulators can generate diagnostic data for application with data-driven techniques [21,22]. While these methods are powerful and enable both detection and identification in systems that rarely fail and thus have little to no failure data, they may require a significant modeling effort, particularly for large-scale systems.

The present effort used the contribution-based approach, but developed a model that served as an alternative to the PCA and DPCA models. This was motivated by the desire to bypass the selection of the latent size. To assess our developed approach, we included the DPCA model in our comparative models.

## 3. Methods

The LOVO anomaly detection and identification approach is implemented by creating a LOVO reconstruction model, which takes measurement data as input, masks key information when making predictions, and tries to recover the input data from the masked data. This model then creates an error term usable for anomaly detection. When the error term exceeds the specified threshold, identification begins, the goal being to estimate which sensors are contributing to the anomaly.

### 3.1. System Model

Time-series data can be modeled as a sequence of measurement vectors $x_{t}\in R^{m}$, each consisting of *m* sensor measurements at sample times t∈{1,2,⋯,n}, where *n* is the number of time steps in the dataset. Because measurements in dynamic processes may be autocorrelated (i.e., depend on information contained in previous sample times), the augmented measurement vector can, for convenience, be defined as: $z_{t}={\left[\begin{array}{ccc} x_{t-s+1}^{T} & \cdots & x_{t}^{T} \end{array}\right]}^{T},$ which includes both the measurement vector for that sample time and the measurement vectors for a fixed window of previous sample times, and where *s* is the window size.

To set up the model, the augmented data samples $z_{t}$ are separated into input and output matrices. This is carried out for each variable, in rotation. A traditional regression approach can then be taken, using the input matrices to predict the output matrices. As the method’s name suggests, a model is created that leaves out one variable from the input data, then uses regression to predict the value of that left-out variable. The input data include all the $z_{t}$-captured time steps in the window. For the output data, only the center sample time is used (meaning the window size should be odd). This approach of only using the center point was selected because some variables cause future changes in others. For example, in a tank system with a heater, turning on the heater will eventually affect the temperature, but will spark almost no immediate change. Therefore, if the model is aimed at predicting the heater power, it would likely be more accurate if it had access to temperature values that were measured both before and after the time stamp of the center point.

The input and output matrices are defined for each variable *i* in the vector $x_{t}$. The matrix definitions are simplified by using the notations $x_{i,t}$ and $x_{-i,t}$. Respectively, these represent variable *i* of $x_{t}$, and all variables in $x_{t}$ apart from variable *i*. Note that when used with $z_{-i,t}$, this means that all instances (in the window) of variable *i* are removed. In addition, $r=\frac{s-1}{2}$ represents the half window size, used to achieve the center sample time. The input and output matrices for variable *i* are then defined as:

(1) $$X_{i}=\left[\begin{array}{ccc} x_{-i,1}^{T} & \cdots & x_{-i,s}^{T}\\ \vdots & \ddots & \vdots \\ x_{-i,n-s+1}^{T} & \cdots & x_{-i,n}^{T} \end{array}\right]=\left[\begin{array}{c} z_{-i,s}^{T}\\ \vdots \\ z_{-i,n}^{T} \end{array}\right],$$

(2) $$Y_{i}=\left[\begin{array}{c} x_{i,s-r}^{T}\\ \vdots \\ x_{i,n-r}^{T} \end{array}\right]=\left[\begin{array}{c} z_{s}^{T}\\ \vdots \\ z_{n}^{T} \end{array}\right]J_{i}^{T},$$

where $X_{i}\in R^{n-s+1\times (m-1)s}$ is the input matrix and $Y_{i}\in R^{n-s+1\times 1}$ is the output matrix, $J=\left[\begin{array}{ccc} 0 & I & 0 \end{array}\right]\in R^{m\times ms}$ selects just the center sample time in the window, the $0\in R^{m\times mr}$ terms in *J* are zero matrices, $I\in R^{m\times m}$ is the identity matrix, and $J_{i}$ is row *i* of *J* and is used to select only variable *i* of that center sample time. Using these matrices, each row of $X_{i}$ serves as the input information applied to predict each row of $Y_{i}$.

Once the input and output matrices are created, they can be used to train a linear regression model for each variable *i*. The regression model is of the following form:

(3) $$J_{i}{\hat{z}}_{t}=A_{i}z_{-i,t}+B_{i},$$

where $A_{i}\in R^{1\times (m-1)s}$ and $B_{i}\in R$ are model coefficients, and ${\hat{z}}_{t}$ is an estimate of $z_{t}$. The unknown coefficients are solved for via ordinary least-squares methods or any of their variants and extensions. For the present effort, ridge regression was used, which places a penalty on the coefficient magnitudes so as to prevent overfitting the model to the data [23]. The penalty magnitude was selected using cross validation.

Once all the model coefficients are solved for, the goal is to then transform the set of models into a single reconstruction model. Using matrix algebra, all these models for individual variables can be combined into a single model of the following form:

(4) $$J{\hat{z}}_{t}=Az_{t}+B,$$

(5) $$A=\left[\begin{array}{ccccc} 0_{1,1} & A_{1,2} & \cdots & A_{1,m-1} & A_{1,m}\\ A_{2,1} & 0_{2,2} & \cdots & A_{2,m-1} & A_{2,m}\\ \vdots & \vdots & \ddots & \vdots & \vdots \\ A_{m-1,1} & A_{m-1,2} & \cdots & 0_{m-1,m-1} & A_{m-1,m}\\ A_{m,1} & A_{m,2} & \cdots & A_{m,m-1} & 0_{m,m} \end{array}\right],$$

(6) $$B={\left[\begin{array}{ccc} B_{1} & \cdots & B_{m} \end{array}\right]}^{T},$$

where $A\in R^{m\times ms}$, $A_{i,j}\in R^{1\times s}$ is the subset (pertaining to the *s* window values) of $A_{i}$ that is multiplied with variable *j*, $0_{i,i}\in R^{1\times s}$ is the zero matrix, and $B\in R^{m}$.

The window size is a crucial hyperparameter for optimizing the performance of this model. This hyperparameter is also frequently used in the other comparative methods examined in the present study. A significant challenge in the selection process for these comparative methods is that the optimal latent size is likely dependent on the window size, as increasing the window size enlarges the input size that must be compressed. Consequently, standard hyperparameter optimization techniques like cross-validation become difficult to apply because the reconstruction error in models such as PCA or AE would vary with the input size, thus preventing an even comparison.

In contrast, the LOVO framework can utilize the standard cross-validation process to select this hyperparameter, as the output remains a fixed size. As a result, cross-validation can be employed to determine the window size that maximizes the prediction error on a validation set. This capability enables a robust data-driven approach to selecting this hyperparameter, enhancing the model’s overall performance.

Returning to the DPCA model introduced in Section 2, the LOVO model can be qualitatively compared with the DPCA model by examining the mechanisms through which they learn underlying data correlations. The primary distinction lies in their model structures. In the LOVO model, the main transformation (5) is a block matrix with zero matrices along the diagonal, which masks key information that the model must then recover from other variables. In contrast, the DPCA transformation does not involve masking but instead forces the model to learn dependencies through the use of a reduced-rank matrix. Both mechanisms allow the respective models to learn correlations. However, a benefit of the LOVO model is that it achieves this without the potentially challenging task of tuning the latent size parameter.

### 3.2. Anomaly Detection

For anomaly detection, the model can be used to generate an anomaly score, which is a scalar value that quantifies the degree to which the augmented data are abnormal. When the score exceeds a certain threshold, the sample is considered anomalous.

To calculate the score, the prediction error $e_{t}$ (often called the residual) is first calculated as the difference between the measured and the estimated values:

(7) $$e_{t}=Jz_{t}-J{\hat{z}}_{t}=\left(J-A\right)z_{t}-B.$$

The score $\phi_{t}$ is then calculated as the weighted sum of the squared error:

(8) $$\phi \left(z_{t}\right)=e_{t}^{T}{\hat{\Sigma }}^{-1}e_{t},$$

where $\hat{\Sigma }$ is a diagonal matrix containing the estimated variances of the error vector that normalizes the error statistics. The anomaly score can be expanded by substituting (7) into (8), then renaming the three constant terms:

(9) $$\phi \left(z_{t}\right)=z_{t}^{T}\Phi_{1}z_{t}-2z_{t}^{T}\Phi_{2}+\Phi_{3},$$

where $\Phi_{1}={\left(J-A\right)}^{T}{\hat{\Sigma }}^{-1}\left(J-A\right)$, $\Phi_{2}={\left(J-A\right)}^{T}{\hat{\Sigma }}^{-1}B$, and $\Phi_{3}=B^{T}{\hat{\Sigma }}^{-1}B$. This expanded version is used to derive the anomaly identification equations.

To calculate the threshold, the error is assumed to be normally distributed, with zero mean and variance ($e_{t}∼N\left(0,\Sigma \right)$). Based on this distribution with estimated variance, the anomaly score follows Hotelling’s $T^{2}$ distribution (i.e., $\phi \left(z_{t}\right)∼T_{p,n-1}^{2}$, where *n* is the number of samples used to estimate the variance and *p* is the number of variables) [24]. The threshold (sometimes called a control limit) is then calculated using the inverse cumulative distribution function, which calculates an upper bound at which the probability of sampling a value less than or equal to the bound equals a user-specified probability. In other words, it provides an upper bound (threshold) that equates to a theoretical probability of declaring a false alarm. The user-specified probability is often called a significance level.

### 3.3. Anomaly Identification

The model is then used to generate reconstruction-based contributions that identify the extent to which each variable contributes to an elevated anomaly score. When an anomaly score exceeds the threshold, this means the model no longer predicts the original data well. As such, the idea is to modify the data until they can be better predicted by the model (i.e., they better follow the patterns learned from normal process behavior). This analysis is accomplished by calculating a sequence of fault-direction matrices and magnitude vectors that modify the original data until the anomaly score falls below the threshold. This section was derived from work contained in [2,5], which uses the PCA model, but was modified to accommodate the structure of the LOVO model.

The fault-direction matrix determines which variables get modified. It is composed of columns of the identity matrix $\xi_{i}$, and the number of columns determines how many variables are modified. For example, in a system with three variables, the fault-direction matrix $\Xi_{t}=\left[\begin{array}{cc} 1 & 0\\ 0 & 0\\ 0 & 1 \end{array}\right]$ comprised of $\xi_{1}$ and $\xi_{3}$ would imply that the first and third variables are modified (i.e., that the contributions to the anomaly stem from these variables), but not the second. The magnitude vector contains a magnitude value corresponding to each direction in the fault-direction matrix.

Using these matrices, the reconstruction-based contribution problem can be framed as an optimization problem in order to determine the fault direction and magnitude that reduce the anomaly score back below the threshold. The fault direction and magnitude are used to define the modified measurement vector $z_{t}-\Xi_{t}f_{t}$, where $f_{t}$ is the fault magnitude. Temporarily assuming a known fault-direction matrix, the fault magnitude is calculated as the solution to the following optimization problem:

(10) $$\arg\min_{f_{t}}\phi \left(z_{t}-\Xi_{t}f_{t}\right).$$

In other words, for a given fault-direction matrix, the solution to this optimization problem finds the corresponding magnitude that modifies the data so that they align as closely as possible with the patterns learned from normal process behavior. The function ϕ(·) is a matrix quadratic function and thus has a unique minimum value that can be calculated by setting its first derivative equal to zero [2]. The solution then becomes:

(11) $$f_{t}={\left(\Xi_{t}^{T}\Phi_{1}\Xi_{t}\right)}^{†}\left(\Xi_{t}^{T}\Phi_{1}z_{t}-\Xi_{t}^{T}\Phi_{2}\right),$$

where ${\left(\cdot \right)}^{†}$ denotes the Moore–Penrose pseudoinverse of a matrix. From this, the contributions of each variable can be calculated as $\Xi_{t}f_{t}$.

The reconstruction-based contribution method can be implemented as either an unsupervised or a supervised algorithm. If it were implemented as a supervised algorithm, there would already exist known fault directions corresponding to known anomalies, and each direction would be used to solve (10) until a solution was found that produced an anomaly score that falls under the threshold (i.e., a viable solution). These directions do not exist with unsupervised anomaly identification; instead, the idea is to find the simplest solution (i.e., the one with the fewest number of compromised variables) that results in such an anomaly score [5].

This unsupervised approach is implemented via combinatorial optimization, in which all the simplest directions (i.e., single-variable directions) are tested first. If none of these directions work, then all directions involving two variables are tested. This process continues until a viable solution is found. This set is defined by an *n*-choose-*k* problem, where *n* is the number of total variables and *k* is the number of candidate variables. Note that this is a slight change from the algorithm outlined in [5]. In that work, the authors proposed that, after calculating all scores for a given number of candidate variables, if no viable solution is found, then the best of those candidate solutions should be the starting point and the next round of candidates would all include that best candidate and add only one extra direction. By contrast, here, all combinations are searched each time the number of candidate variables is incremented. While this proposed change could increase the computational cost because of the additional combinations, it showed better performance in the controlled datasets and was thus adopted here. For datasets with many variables, the original approach may become necessary.

When used with the original PCA model and window sizes greater than one, the algorithm employed a different vector in the fault-direction matrix for each time step in the window and for each variable. This resulted in *s* candidate directions for each variable. Unfortunately, this strategy does not work for the LOVO model. The problem is that because the LOVO model only predicts the center value within each window, the identification algorithm has *s* degrees of freedom for each variable added in the combinatorial optimization problem to minimize the prediction error of *m* variables, quickly resulting in an underconstrained optimization problem. To overcome this, we used an approximation that causes the estimated anomaly magnitude to remain unchanged over the duration of the window. Mathematically, this can be implemented by stacking columns of the identity matrix on top of each other, resulting in, for example, $\xi_{1}={\left[\begin{array}{cccccc} 1 & 0 & 0 & 1 & 0 & 0 \end{array}\right]}^{T}$ for a three-variable model with a window size of two.

## 4. Data

To demonstrate the proposed approach, the present study used simulated data based on an SMD system and open-source experimental data from the SKAB dataset [6].

### 4.1. Spring–Mass–Damper Data

The basic building blocks of the SMD system are springs, masses, dampers, sensors, and actuators: the masses respond to forces, the springs apply restorative forces to the mass proportional to the spring displacement, the dampers apply damping forces to the mass proportional to the damper velocity, the actuators apply forces directly to masses, and the sensors measure the positions of the masses [25].

The SMD system was selected for this research for several reasons. First, though it is conceptually simple and features just a few basic components, those components can be combined to generate high-order systems with coupled variables. Second, the system is easily scalable to include many sensors and actuators. Third, it allows for the straightforward incorporation of sensor anomalies (by directly modifying sensor measurements) and process anomalies (by modifying system parameters—here, the spring stiffness and damping coefficients).

**Figure 2:**
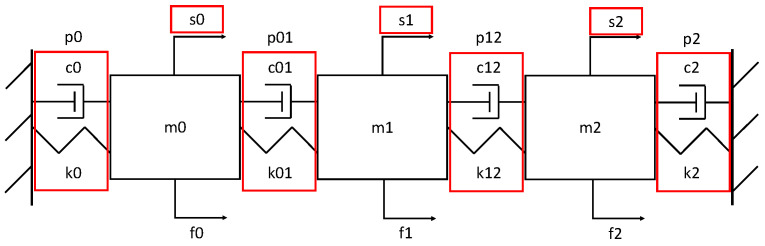
A sketch of the SMD system, with anomaly types highlighted in red.

**Figure 3:**
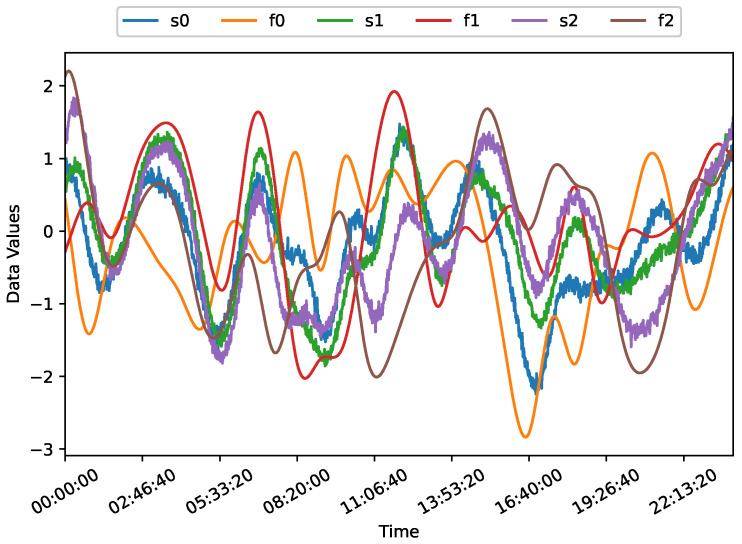
Time-series data for the SMD position sensors and actuators.

This study used a system with three masses (labeled m), four springs and dampers (labeled k and c, respectively), and two fixed ends, as shown in Figure 2. All connections used linear spring and damper components, meaning that their forces were linearly proportional to their relative displacements and velocities, respectively. In addition, position sensors (labeled s) and actuators that applied forces (labeled f) were placed on each mass. This system is governed by the following differential equations:

(12) $$\begin{array}{c} M\ddot{x}+C\dot{x}+Kx=f, \end{array}$$

(13) $$\begin{array}{c} M=\left[\begin{array}{ccc} m_{0} & 0 & 0\\ 0 & m_{1} & 0\\ 0 & 0 & m_{2} \end{array}\right], \end{array}$$

(14) $$\begin{array}{c} C=\left[\begin{array}{ccc} c_{0}+c_{01} & -c_{01} & 0\\ -c_{01} & c_{01}+c_{12} & -c_{12}\\ 0 & -c_{12} & c_{12}+c_{2} \end{array}\right], \end{array}$$

(15) $$\begin{array}{c} K=\left[\begin{array}{ccc} k_{0}+k_{01} & -k_{01} & 0\\ -k_{01} & k_{01}+k_{12} & -k_{12}\\ 0 & -k_{12} & k_{12}+k_{2} \end{array}\right], \end{array}$$

where $x={\left[\begin{array}{ccc} x_{0} & x_{1} & x_{2} \end{array}\right]}^{T}$ are the displacements of the masses, $\dot{x}={\left[\begin{array}{ccc} {\dot{x}}_{0} & {\dot{x}}_{1} & {\dot{x}}_{2} \end{array}\right]}^{T}$ and $\ddot{x}={\left[\begin{array}{ccc} {\ddot{x}}_{0} & {\ddot{x}}_{1} & {\ddot{x}}_{2} \end{array}\right]}^{T}$ are the first and second derivatives of displacement with respect to time, representing velocity and acceleration, respectively, and $f={\left[\begin{array}{ccc} f_{0} & f_{1} & f_{2} \end{array}\right]}^{T}$ are the forces applied to the masses. Sample time-series data generated via these equations are shown in Figure 3.

Within this system, seven anomaly types were selected (highlighted in red in Figure 2), consisting of both process and sensor anomalies. Here, a process anomaly (labeled p) was defined as an anomaly that changes the physics of the system (e.g., pump wear or failure). Process anomalies in the SMD system occurred on the spring and damper components of a process connection point between either two masses or a mass and a fixed end. A sensor anomaly (labeled s) was defined as an anomaly that changes the measurement data (e.g., a temperature sensor bias or failure that alters the data received). Sensor anomalies in the SMD system occurred in the position sensors. The process and sensor anomalies used in the SMD system are labeled in Table 1.

The anomalies were inserted into the differential equations as ramp functions, meaning the effects of the anomalies (called anomaly magnitude) started at zero, then slowly increased to their maximum effect. As such, in the early stages of each anomaly, the magnitude was so small that the anomaly was impossible to detect. This approach was taken because it emulated anomalies of many different magnitudes, providing more opportunities to distinguish between superior and inferior algorithms (under the assumption that better algorithms will detect anomalies earlier in the ramp function).

To further emulate real systems, this simulation also accounted for both process and measurement noise. Process noise was implemented as semi-periodic and time-varying external forces applied to the system, akin to wind forces. Measurement noise was implemented as random disturbances to the sensor readings, since real sensors cannot perfectly capture the true parameter of interest they are estimating. These two noise sources can be thought of as different types of contamination to assess the robustness of the proposed and comparative methods, with the process noise acting as a semi-periodic contamination approach and the sensor noise acting as a random contamination approach.

For the present study, five datasets were generated, each with the same general SMD structure, but with stochastically selected spring and damper constants, mass values, actuator force sequences, and anomaly types and magnitudes. In addition, multiple tests were conducted with increasing levels of data contamination. Each dataset included six 2-year training sets consisting of 0%, 2%, 4%, 6%, 8%, and 10% anomalous data, and one 50-year testing set containing 1000 anomalies. The lengthy testing set was selected to ensure a rich variety of anomaly types. Each training set was used to train a model, the goal being to assess how method performance was affected by (unknown to the algorithm) anomalies in the training data. The same test set was used with each training set.

### 4.2. Skoltech Anomaly Benchmark Data

The SKAB experimental testbed comprises a water-circulation loop with a water tank, motor-driven pump, two solenoid valves, and various sensors. These sensors measured water flow and pressure, pump vibration and temperature, and motor current and voltage. Additionally, the dataset included labels for normal and anomalous conditions based on known changes to the system (see [6] for more details).

In this study, all data from the Valve 1 and Valve 2 experiments were utilized. The normal data were divided in half; the first half was used for training, and the second half for testing. Similar to the SMD datasets, data contamination was considered by adding the same percentages of anomalous data to the training set.

## 5. Assessment Metrics

Given the number of datasets and the anomalies within them, it was necessary to condense the outputs of the detection and identification algorithms into a set of assessment metrics. These metrics summarize the methods’ performance and can be used for comparison purposes. In this effort, the assessment metrics measured anomaly detection and identification performance separately.

### 5.1. Anomaly Detection

Many anomaly detection methods are comprised of two parts: a scoring algorithm that assigns an anomaly score to a data sample, and a classifier that uses that score to classify the data as either normal or anomalous (e.g., scores above a given threshold are classified as anomalous; those below are classified as normal). Because the scoring algorithm is inherent in the classifier, the classifier performance can be used to assess the detection methods. This section uses the following conventions from the classification literature: positive (P), predicted positive (PP), true positive (TP), false positive (FP), negative (N), predicted negative (PN), true negative (TN), and false negative (FN). Here, a time-series sample is classified positive if it is anomalous, and negative if it is normal.

The selected assessment metric needed to be independent of two factors: the ratio of normal to anomalous data, and the threshold used to classify whether data are normal or anomalous. Starting with the first factor, a common classification assessment metric is accuracy, defined as (TP + TN)/(P + N). The challenge with this metric is that, for example, if the data consist of 990 negative samples and 10 positive ones (i.e., 99% normal), a classifier that predicts all data as normal would give an accuracy of 99%. However, this classifier is obviously not useful when it is important to detect the anomalies within the data.

To overcome this first factor, the present effort used precision and recall rather than accuracy. Precision and recall—defined as TP/(TP + FP) and TP/(TP + FN), respectively—are commonly used to report metrics for imbalanced classification [26]. Returning to the example above, the precision would be undefined, and the recall would be 0%, clearly showing that the classifier is working poorly. The above example (Example 1), along with a second classifier (Example 2) that labels everything as being positive, are shown in Table 2.

Moving to the second factor, using the examples from Table 2, Example 1 is equivalent to selecting a threshold above the maximum score (i.e., naively classifying everything as normal), and Example 2 is equivalent to selecting a threshold below the minimum score (i.e., naively classifying everything as anomalous). In both examples, the scoring algorithm is completely ignored by the classifiers. This shows that precision and recall are very sensitive to the threshold selection method. During actual detection in production, a threshold must be selected, but this is less important when comparing methods against each other.

To overcome this second factor, a metric called the precision–recall area under the curve (PR-AUC) was used. Parameterized by varying threshold values, precision and recall can be plotted against each other to show the tradeoff between them. The PR-AUC metric then calculates the area under this curve, thereby removing the threshold from the calculation entirely.

### 5.2. Anomaly Identification

Given that this effort focused on unsupervised anomaly identification, a concept of accuracy does not necessarily apply when it comes to identification. Rather, it is more relevant to consider how useful the diagnostic information would be to an SME. This being the case, we considered two questions in assessing identification results: how interpretable and how repeatable are they? The interpretability question, though subjective, is a necessary consequence of using unsupervised approaches. Because anomaly types are highly dependent on the system of interest and there is a lack of previously labeled anomaly data, SMEs will always be required to interpret the results. The repeatability question is more quantifiable and is made necessary because the results are stochastic, meaning they are a function of noise and uncertainty. As such, the results should be examined over multiple instances of each possible anomaly type.

The results of the identification algorithm were sequences of contribution values for each variable, which, for assessment purposes, were transformed into anomaly directions by summing the contributions of each variable over the duration of the anomaly (ignoring windows in which the anomaly score was below the threshold), then normalizing the sums to make them into unit vectors. Mathematically, this was calculated via:

(16) $$TC_{i}=\frac{\sum_{t=t_{1}}^{t_{2}}C_{i,t}}{\sqrt{\sum_{i=1}^{m}(\sum_{t=t_{1}}^{t_{2}}C_{i,t}}{)}^{2}},$$

where $C_{i,t}$ is the contribution of variable *i* at sample time *t*, $TC_{i}$ is the total contribution of variable *i*, $t_{1}$ is the start time of the anomaly, and $t_{2}$ is the end time of the anomaly. The total contributions for all the variables were then concatenated into a vector. As an example, Table 3 shows the anomaly direction vector for an s1 sensor anomaly.

To assess interpretability, the individual anomaly directions, grouped by anomaly type, were averaged to determine the typical anomaly direction for that category. If the average direction for a given type was such that an SME could correctly identify the components involved in the anomaly, the anomaly was said to be interpretable.

Repeatability assessed how distinct the average directions for each anomaly type were, and whether enough variation existed within each type to cause potential misclassification. These assessments were conducted using a classification rule based on the cosine similarity metric, which measures the similarity between two vectors. Given the vectors *a* and *b*, the cosine similarity is defined as (see, for example, [27]):

(17) $$\cos\theta =\frac{a\cdot b}{∥a∥∥b∥},$$

where · is the dot-product operator, ∥v∥ is the magnitude of a vector *v*, and θ is the angle between vectors *a* and *b*. Assuming that the vector components are non-negative, this metric will equal one when *a* and *b* are colinear, and zero when they are orthogonal. Consequently, the further from one that this metric is, the more dissimilar the given vectors can be said to be. Cosine similarity can be applied as a classifier in the following manner:

1. Calculate average anomaly directions for each anomaly type in the dataset.
2. For each anomaly in the dataset, find the cosine similarity between that specific anomaly’s direction and the average direction for each anomaly type.
3. Classify the specific anomaly as the type associated with the highest cosine similarity score.

## 6. Results

For detection, the LOVO model was implemented on the SMD and SKAB datasets over six levels of data contamination. The LOVO model detection performance was compared against PCA, AE, and iForest models, with the iForest model utilizing PCA for feature extraction. The iForest algorithm was also tested without PCA, but had poor performance.

As noted, one challenge with the comparative models is selecting an appropriate latent dimension. To ensure the reported results reflect the approach rather than the latent size selection process, the reported results use the optimal latent size for each method. However, we additionally conducted a sensitivity analysis by calculating the detection performance across a range of latent spaces.

For identification, the LOVO model was only implemented on the SMD datasets and only against the PCA model due to the limited availability of unsupervised identification algorithms.

### 6.1. Anomaly Detection

Table 4 presents the detection results obtained from the four methods. For the SMD dataset, this was summarized as the average over the five datasets plus or minus one standard deviation. For the SKAB dataset, this was the performance of the one test set used. Moreover, the highest-performing method for each level of data contamination is highlighted in bold. Per analysis of these results for the SMD dataset, the PCA method slightly outperformed the LOVO method when there were no data contamination, but the LOVO method performed best in all scenarios in which there were data contamination. Furthermore, the reduction in performance due to data contamination was notably greater for the comparative methods than for the LOVO method. The results of the SKAB dataset show a combination of PCA and iForest performing best in all scenarios.

**Figure 4:**
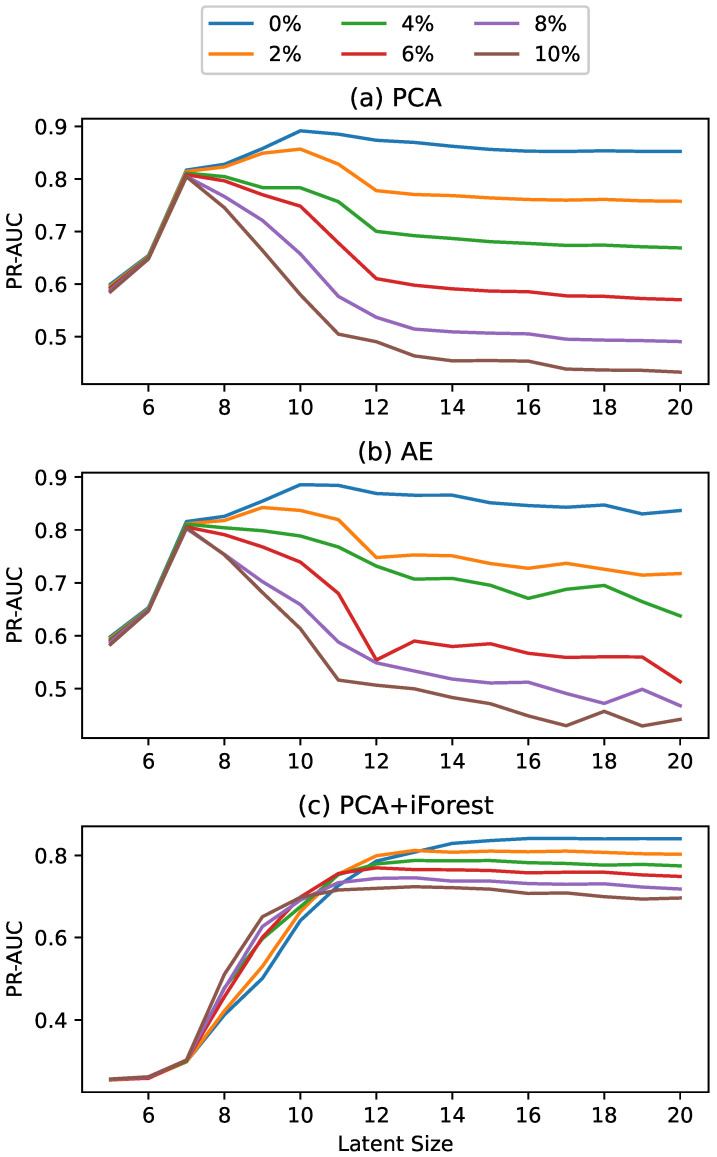
Anomaly detection results for the SMD dataset showing the PR-AUC for the (**a**) PCA, (**b**) AE, and (**c**) iForest models, parameterized by both latent size and the level of data contamination.

**Figure 5:**
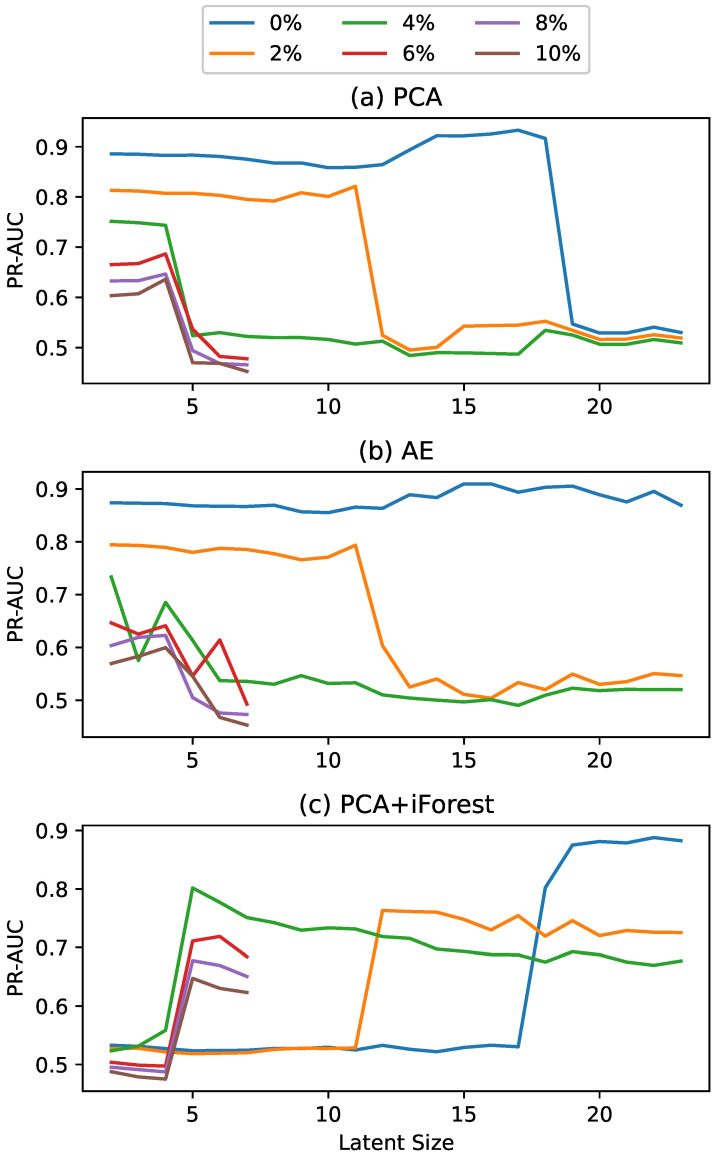
Anomaly detection results for the SKAB dataset showing the PR-AUC for the (**a**) PCA, (**b**) AE, and (**c**) iForest models, parameterized by both latent size and the level of data contamination.

As mentioned previously, the performances of the comparative methods were dependent upon the latent-size selection, with the above results reflecting the use of the optimal size. To quantify this dependency, PR-AUC was calculated for each of the comparative methods and levels of data contamination as a function of latent size. This relationship is illustrated in Figure 4 and Figure 5 for the SMD and SKAB datasets, respectively. In the SMD dataset results illustrated in Figure 4, the window size was sufficiently large across all levels of data contamination such that the maximum tested latent size exceeded those displayed in the figure. For the SKAB dataset results illustrated in Figure 5, the window size was smaller and varying, resulting in different maximum tested latent sizes across the levels of data contamination.

Starting with the SMD sensitivity analysis, the PCA and AE models with little to no data contamination showed superior performance at higher latent sizes; however, as contamination increased, their performances at those higher latent sizes quickly dropped, showing much more sensitivity to this parameter. By way of an explanation, we believe that when there is no contamination in the training data, a larger latent size can be selected that captures the more subtle aspects of the dynamics, resulting in a higher level of performance. However, when there are anomalies, some of those latent dimensions will contain anomalous patterns. Therefore, including more latent dimensions means that the model learns those anomalous patterns that do not generalize. In contrast, the iForest algorithm performed very differently, with worse performance for lower latent sizes and superior performance for higher latent sizes. However, regardless of latent size, the iForest performed worse than the other methods investigated.

Examining the SKAB sensitivity analysis, a similar trend is observed. The PCA and AE models exhibited less sensitivity to latent size with minimal data contamination; however, as contamination levels increased, their sensitivity to latent size increased significantly. The iForest algorithm generally showed superior performance at higher latent sizes, though there was still some performance decrease when deviating from the optimal latent size.

Based on these sensitivity analyses, it is evident that these methods are sensitive to latent size, and this sensitivity generally intensifies with increasing data contamination.

### 6.2. Anomaly Identification

**Figure 6:**
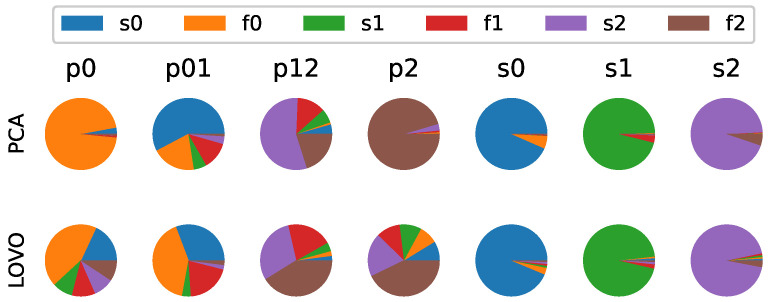
Anomaly identification results showing the average directions, parameterized by method and anomaly type.

For the identification task, interpretability and repeatability were assessed separately. The interpretability results for the SMD dataset are presented as pie charts for both methods and seven different anomaly types (Figure 6), and the results were interpreted in detail.

Starting with the p0 process anomaly type, the contributions for the PCA method showed up almost entirely in the f0 variable. By contrast, those for the LOVO method showed up primarily in the f0 variable, though a large portion was also contained in the s0 variable and smaller portions in the remaining variables. Returning to the system schematic in Figure 2, the connections between m0 and the fixed end can only apply a force to m0; thus, it makes sense that an anomaly in those components would show up as an anomaly in the f0 variable. Based on these results, the contributions for the PCA method make the most sense, though those for the LOVO method are also intuitive to interpret.

The next type is the p01 process anomaly. Unlike the average contributions for the p0 process anomaly type, these average contributions showed a greater mixture of variables for both methods, with contributions primarily coming from s0, f0, s1, and f1 (though in different proportions for each of the two methods). Given that this anomaly specifically occurs between m0 and m1, the results of both methods were intuitive and would provide insight to SMEs. Due to system symmetry, the p2 and p12 process anomaly types were mirror images of the p0 and p12 types, respectively, and would thus indicate similar conclusions.

Finally, the interpretability results generated for the different sensor anomaly types can be assessed. The results for all three sensor anomaly types and both methods showed similar results, with their contributions showing up predominantly in their respective variables. As such, these average contributions were interpretable as well.

Moving to repeatability, Table 5 shows the classification results for the two methods. Per these results, the PCA methods showed better repeatability than the LOVO method, across all training cases. That being said, both methods showed excellent repeatability, suggesting them to be fairly robust to noise and uncertainty. One likely cause of the LOVO method’s slightly inferior performance is that the LOVO’s p0 and p2 average directions were more diverse than those of the PCA method, making them appear for the LOVO method more similar to the p01 and p12 average directions, respectively.

## 7. Conclusions

This study presented a comprehensive evaluation of the newly developed LOVO model for anomaly detection and identification. The LOVO method was applied to synthetic data generated from an SMD simulator and experimental data from the SKAB dataset, and the results were compared against PCA, AE, and iForest models.

Regarding detection using the SMD dataset, the LOVO method performed better than the comparative method in all scenarios with data contamination, and performed only slightly worse than the PCA method when no contamination was present. Using the SKAB dataset, the comparative methods outperformed the LOVO model regardless of data contamination level. However, these conclusions for the comparative methods pertain to using the optimal latent size, which may not always be feasible to select. In addition, the comparative methods all showed sensitivity to a poor choice of latent size, particularly in the presence of data contamination. In contrast, the LOVO approach avoids any comparably sensitive hyperparameter that must be selected.

As regards identification, this study only used the SMD dataset and looked at both the interpretability and repeatability of the results. Starting with interpretability, both methods produced interpretable results, though the PCA method’s results were slightly more so. This performance trend held true for repeatability as well, with the PCA method producing more repeatable results regardless of the amount of anomalies in the training data. Nevertheless, the LOVO method’s identification results were also highly repeatable.

These findings make it evident that the LOVO approach can serve as a powerful model in regard to both detection and identification, and its lack of a latent dimension makes it a potentially more straightforward modeling approach when automating the creation of process anomaly detection models for large-scale systems featuring a large number of sensors and components. That said, there are some limitations to consider. The primary limitation is that although training a small number of these models is computationally inexpensive, scaling up the process to an entire large-scale system significantly increases the computational cost. This additional computational cost is only associated with the training process; however, once trained, the models are comparable in terms of computational cost for running inferences.

The LOVO approach (as presented herein) focuses on using linear methods and models for anomaly detection and identification. These can perform well when paired with dynamic systems that exhibit predominantly linear behavior. However, in real life, many systems have nonlinearities, and ignoring them could cause significantly degraded performance. Thus, future work will look to extend these methods by incorporating nonlinear models into the framework.

## 8. Disclaimer

Idaho National Laboratory is a multi-program laboratory operated by Battelle Energy Alliance, LLC, for the U.S. Department of Energy under contract DE-AC07-05ID14517. This work of authorship was prepared as an account of work sponsored by an agency of the U.S. Government. Neither the U.S. Government, nor any agency thereof, nor any of their employees makes any warranty, express or implied, or assumes any legal liability or responsibility for the accuracy, completeness, or usefulness of any information, apparatus, product, or process disclosed, or represents that its use would not infringe privately owned rights. The publisher, by accepting the article for publication, acknowledges that the U.S. Government retains a nonexclusive, paid-up, irrevocable, worldwide license to publish or reproduce the published form of this manuscript, or allow others to do so, for U.S. Government purposes. The views and opinions of authors expressed herein do not necessarily state or reflect those of the U.S. Government or any agency thereof. The document number issued by Idaho National Laboratory for this paper is INL/JOU-24-79565.

## Figures and Tables

**Table 1:** Descriptions of the seven different anomaly types.

Type	Description
p0	Process anomaly that modifies the spring and damper coefficients between $m_{0}$ and the left end.
p01	Process anomaly that modifies the spring and damper coefficients between $m_{0}$ and $m_{1}$.
p12	Process anomaly that modifies the spring and damper coefficients between $m_{1}$ and $m_{2}$.
p2	Process anomaly that modifies the spring and damper coefficients between $m_{2}$ and the right end.
s0	Sensor anomaly that modifies the $s_{0}$ sensor data.
s1	Sensor anomaly that modifies the $s_{1}$ sensor data.
s2	Sensor anomaly that modifies the $s_{2}$ sensor data.

**Table 2:** Example accuracy, precision, and recall of two classifiers for an imbalanced dataset.

Example	PP	PN	TP	TN	FP	FN	Accuracy	Precision	Recall
1	0	1000	0	990	0	10	0.99	Undefined	0
2	1000	0	10	0	990	0	0.01	0.01	1

**Table 3:** Example summary statistics (normalized to be a unit vector, then rounded) for an s1 sensor anomaly.

s0	f0	s1	f1	s2	f2
0.02	0.02	1.0	0.0	0.01	0.01

**Table 4:** Anomaly detection results showing the PR-AUC, parameterized by both method and the level of data contamination. The bold highlighting indicates the highest-performing method for each level of data contamination.

Dataset	% Cont.	Methods			
		LOVO	PCA	AE	PCA + iForest
SMD	0	0.886 ± 0.002	**0.892** ± **0.001**	0.886 ± 0.002	0.843 ± 0.007
	2	**0.873** ± **0.005**	0.857 ± 0.021	0.846 ± 0.021	0.818 ± 0.012
	4	**0.864** ± **0.004**	0.820 ± 0.015	0.818 ± 0.013	0.793 ± 0.007
	6	**0.857** ± **0.003**	0.815 ± 0.013	0.813 ± 0.012	0.770 ± 0.009
	8	**0.852** ± **0.008**	0.812 ± 0.013	0.808 ± 0.010	0.747 ± 0.006
	10	**0.847** ± **0.009**	0.804 ± 0.012	0.805 ± 0.012	0.729 ± 0.007
SKAB	0	0.866	**0.933**	0.910	0.888
	2	0.792	**0.821**	0.794	0.763
	4	0.730	0.752	0.734	**0.802**
	6	0.674	0.687	0.647	**0.719**
	8	0.635	0.647	0.623	**0.678**
	10	0.597	0.636	0.600	**0.647**

**Table 5:** Anomaly identification results showing the classification accuracy, parameterized by both method and the level of data contamination. The bold highlighting indicates the highest-performing method for each level of data contamination.

% Contamination	Methods	
	LOVO	PCA
0	0.970 ± 0.033	**0.996** ± **0.002**
2	0.973 ± 0.017	**0.991** ± **0.007**
4	0.974 ± 0.018	**0.989** ± **0.015**
6	0.978 ± 0.023	**0.994** ± **0.012**
8	0.978 ± 0.019	**0.990** ± **0.021**
10	0.937 ± 0.057	**1.000** ± **0.000**

## Data Availability

The SMD dataset supporting the conclusions of this article will be made available by the authors on request. The SKAB dataset is available at the website shown in the References.
